# Assessing the accuracy of a new 3D2D registration algorithm based on a non-invasive skin marker model for navigated spine surgery

**DOI:** 10.1007/s11548-022-02733-w

**Published:** 2022-08-20

**Authors:** Bas J. J. Bindels, Rozemarijn A. M. Weijers, Martijn S. van Mourik, Robert Homan, Jan J. Rongen, Maarten L. J. Smits, Jorrit-Jan Verlaan

**Affiliations:** 1 Department of Orthopedic Surgery, University Medical Center Utrecht – Utrecht University, Heidelberglaan 100, 3584 CX Utrecht, The Netherlands; 2 Department of Radiology, University Medical Center Utrecht – Utrecht University, Heidelberglaan 100, 3584 CX Utrecht, The Netherlands; 3 Philips Healthcare, Veenpluis 6, 5684 PC Best, The Netherlands

**Keywords:** Surgical navigation, Image-guidance, Computer-assisted surgery, Fluoroscopy, Spine, Vertebra

## Abstract

**Purpose:**

We assessed the accuracy of a new 3D2D registration algorithm to be used for navigated spine surgery and explored anatomical and radiologic parameters affecting the registration accuracy. Compared to existing 3D2D registration algorithms, the algorithm does not need bone-mounted or table-mounted instruments for registration. Neither does the intraoperative imaging device have to be tracked or calibrated.

**Methods:**

The rigid registration algorithm required imaging data (a pre-existing CT scan (3D) and two angulated fluoroscopic images (2D)) to register positions of vertebrae in 3D and is based on non-invasive skin markers. The algorithm registered five adjacent vertebrae and was tested in the thoracic and lumbar spine from three human cadaveric specimens. The registration accuracy was calculated for each registered vertebra and measured with the target registration error (TRE) in millimeters. We used multivariable analysis to identify parameters independently affecting the algorithm’s accuracy such as the angulation between the two fluoroscopic images (between 40° and 90°), the detector-skin distance, the number of skin markers applied, and waist circumference.

**Results:**

The algorithm registered 780 vertebrae with a median TRE of 0.51 mm [interquartile range 0.32–0.73 mm] and a maximum TRE of 2.06 mm. The TRE was most affected by the angulation between the two fluoroscopic images obtained (*p* < 0.001): larger angulations resulted in higher accuracy. The algorithm was more accurate in thoracic vertebrae (*p* = 0.004) and in the specimen with the smallest waist circumference (*p* = 0.003). The algorithm registered all five adjacent vertebrae with similar accuracy.

**Conclusion:**

We studied the accuracy of a new 3D2D registration algorithm based on non-invasive skin markers. The algorithm registered five adjacent vertebrae with similar accuracy in the thoracic and lumbar spine and showed a maximum target registration error of approximately 2 mm. To further evaluate its potential for navigated spine surgery, the algorithm may now be integrated into a complete navigation system.

**Supplementary Information:**

The online version contains supplementary material available at 10.1007/s11548-022-02733-w.

## Introduction

Minimally invasive spine surgery is associated with better patient outcomes and lower overall costs than open spine surgery [1–5]. During minimally invasive spine surgery, surgeons strongly depend on intraoperative imaging to visualize relevant anatomical structures, and surgical hardware like screws and rods.

Compared to intraoperative two-dimensional (2D) fluoroscopic imaging, intraoperative three-dimensional (3D) navigation has large potential as spine surgeons can place pedicle screws more accurately while maintaining a short operation time, also (or rather, especially) in anatomically challenging cases [6–8]. However, the required 3D imaging and navigational equipment is often heavy, cumbersome, and expensive [9–11].

We developed a new 3D2D registration algorithm for spine surgery that registers vertebrae from a preoperatively acquired CT to an intraoperative situation using 2D fluoroscopic imaging. In future, the new 3D2D registration algorithm may facilitate low-cost and easy-to-use 3D navigation without disrupting the routine fluoroscopic-guided workflow. The algorithm is based on non-invasive hybrid skin markers (radiopaque and optical), which are used to register the navigated optical space to the fluoroscopic space. Compared to existing 3D2D registration algorithms for navigated spine surgery, the algorithm does not need any bone-mounted or table-mounted instruments for registration. Neither does the algorithm require additional equipment attached to the intraoperative fluoroscopic imaging device to calibrate or track the imaging device [8, 12, 13].

In this study, we assessed the accuracy of the new 3D2D registration algorithm based on a non-invasive skin marker model, and explored anatomical and radiologic parameters affecting the registration accuracy.

## Materials and methods

The study was conducted at the Department of Radiology and a surgical suite for experimental surgery of a university affiliated hospital in the Netherlands. The experiments were performed in compliance with the ethical guidelines for human cadaveric studies. All donors had provided written permission that their remains were to be used for research purposes. The study subjects were three fresh-frozen human torsos with no history of spinal surgery (Table 1).

**Table 1:** Characteristics of study subjects

	Subject A	Subject B	Subject C
Gender	Male	Male	Female
Age (years)	88	78	51
Waist circumference (centimeter)	121	96	73
Radiologic findings on baseline CT	Multiple sclerotic lesions throughout the vertebral column, possibly (prostate cancer) metastases Compression fracture 12th thoracic vertebra	None	Multiple lytic lesions 9th and 10th thoracic vertebrae Compression fractures 10th and 11th thoracic vertebrae

### Imaging data, marker model, and 3D2D registration algorithm

A baseline CT scan (Philips Brilliance 64 CT scanner, Philips, Best, Netherlands) was used to obtain 3D data (slice thickness 0.67 mm, contiguous slices, reconstruction matrix 512 × 512).

The (2D) fluoroscopic images for registration were acquired with a mobile C-arm system (Philips Zenition 70, Philips, Best, Netherlands). The imaging settings were set to the spine protocol (variable kV, typical dose-level 0.408 mGy 20 cm PMMA) to achieve optimal image quality of the vertebrae, which was part of the regular software (version 5.1.7: IQ NA HC R5.1.7).

All imaging data files were transferred to a secured portable computer in Digital Imaging and Communications in Medicine (DICOM) format.

**Fig. 1:**
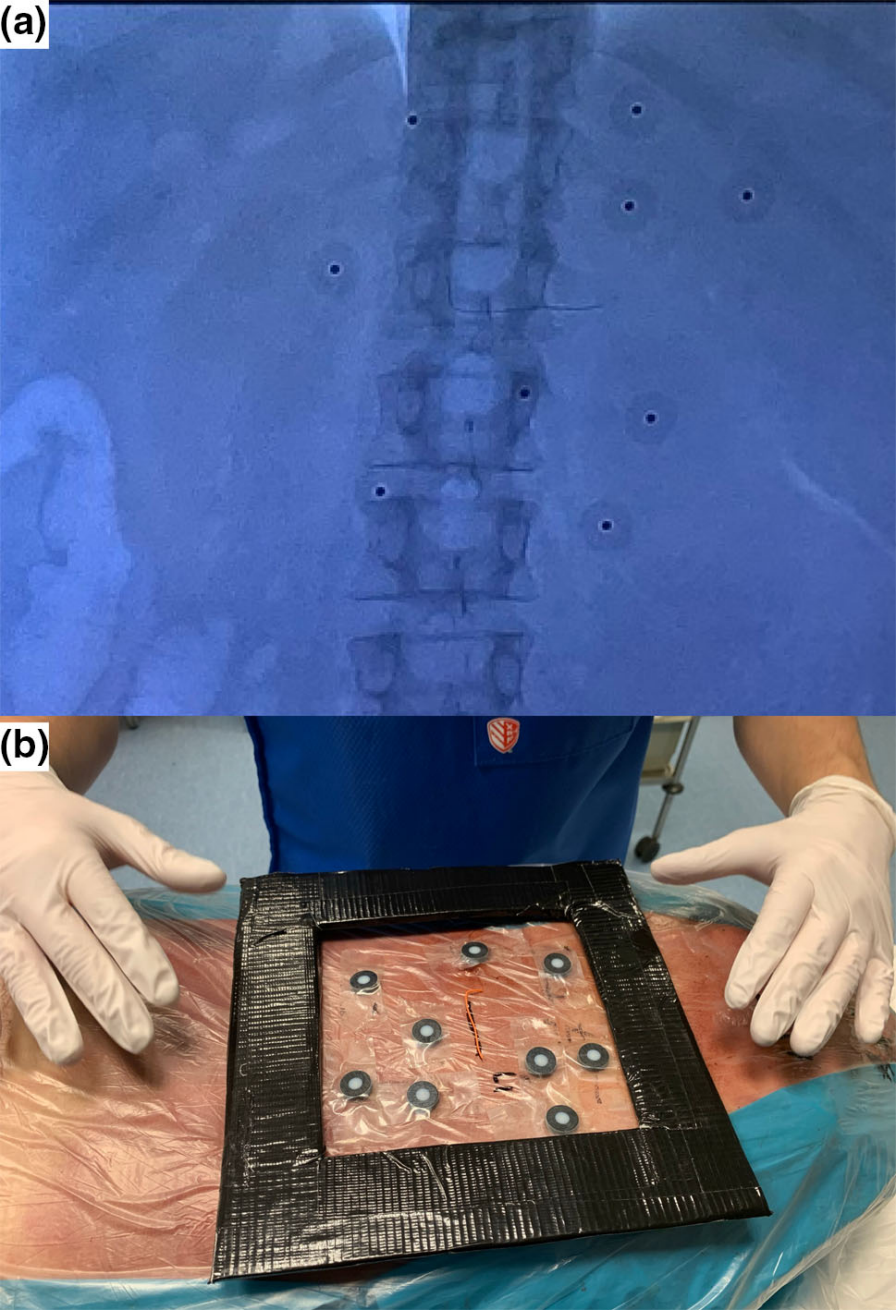
Examples of the hybrid skin markers. **a** Fluoroscopic image capturing nine markers containing a radiopaque sphere, **b** nine markers applied to the skin

The non-invasive marker model consisted of a randomly applied pattern of prototype hybrid skin markers (radiopaque and optical), which were an update of previously used optical markers [14]. The update consisted of a radiopaque sphere added to the marker’s center to make them visible on fluoroscopy (Fig. 1).

The 3D2D registration was performed offline by running image data through a prototype algorithm (Philips Healthcare, Best, the Netherlands) on a regular computer (Intel® Core™ i7-9750H processor, NVIDIA Quadro® T1000 graphics card).

### 3D2D registration process

The prototype 3D2D registration algorithm contains three different functionalities: a segmentation algorithm [15], a registration algorithm, and a pose-estimation algorithm.

After the anatomical level of one vertebra was manually indicated on the baseline CT, the model-based segmentation algorithm automatically segmented all vertebrae present in the CT. Subsequently, the registration algorithm processed the segmented vertebrae into digitally reconstructed radiograph (DRR) images using a forward projection algorithm [16].

Then, the anatomical level of one vertebra was manually indicated on one fluoroscopic image and the registration algorithm matched the segmented vertebrae to their position in the fluoroscopic images (2D) with a rigid registration per vertebrae to correct for inter-vertebrae deformation. The vertebrae were matched based on their gradients, and the gradient differences were used as similarity measure (*S*) to compare the DRRs and fluoroscopic images (Eq. 1):

1 $$ S = \mathop \sum \limits_{i,j} \frac{{A_{{\text{v}}} }}{{A_{v} + \left( {I_{{{\text{diffV}}}} \left( {i,j} \right)} \right)^{2} }} + \mathop \sum \limits_{i,j} \frac{{A_{{\text{h}}} }}{{A_{{\text{h}}} + \left( {I_{{{\text{diffH}}}} \left( {i,j} \right)} \right)^{2} }} $$

2 $$ I_{{{\text{diffV}}}} \left( {i,j} \right) = \frac{{{\text{d}}I_{{{\text{fl}}}} }}{{{\text{d}}i}} - s \frac{{{\text{d}}I_{{{\text{DRR}}}} }}{{{\text{d}}i}} $$

3 $$ I_{{{\text{diffH}}}} \left( {i,j} \right) = \frac{{{\text{d}}I_{{{\text{fl}}}} }}{{{\text{d}}j}} - s \frac{{{\text{d}}I_{{{\text{DRR}}}} }}{{{\text{d}}j}} $$

In Eq. (1), ${A}_{\mathrm{v}}$ represents a constant value for the maximum vertical similarity, and ${A}_{\mathrm{h}}$ a constant value for the maximum horizontal similarity. Equation (1) is structured according to the form $\frac{1}{1+{x}^{2}}$ to normalize the similarity between 0 (minimum similarity) and 1 (maximum similarity) [12, 17]. In Eqs. 2 and 3, the gradient-based measure first differentiates ${I}_{\mathrm{fl}}$ and ${I}_{\mathrm{DRR}}$ and then takes the vertical difference image (${I}_{\mathrm{dif fV}}$) (Eq. 2) or the horizontal difference image (${I}_{\mathrm{dif fH}}$) (Eq. 3). The gradient-based registration optimizes the geometrical position, $q{=({t}_{x},{ t}_{y},{ t}_{z}, {\omega }_{x}, {\omega }_{y}, {\omega }_{z})}^{\mathrm{T}}$, which is the input of the DRR calculation, using the Covariance Matric Adaption Evolution Strategy (CMA-ES) to avoid local minima’s [18].

The registration algorithm executed the 3D2D registration twice. First, using one fluoroscopic image, a rough rigid registration was performed to reduce the search space for the second, more complicated per vertebrae registration based on two fluoroscopic images. During the first registration, two adjacent vertebrae were simultaneously matched.

Before the second registration, the pose-estimation algorithm calculated the pose difference—or relative angulation—between the two fluoroscopic images. The pose-estimation algorithm is an Umeyama-based algorithm which determines the pose based on the 3D model of the radiopaque spheres from the skin markers [19]. The automatic pose-estimation avoided that the absolute angulation and rotation of the mobile C-arm had to be calibrated or that its relative position to the study subject had to be tracked [20]. During the second registration, each vertebra was separately matched to both fluoroscopic images that have a fixed position with respect to each other. Per vertebrae registration corrects for possible spinal curvature changes and shifting of adjacent vertebrae relative to each other that could have occurred between the baseline CT scan and fluoroscopic image acquisition. We assessed the accuracy of the prototype 3D2D registration algorithm based on the second registration.

### Reference standard

**Fig. 2:**
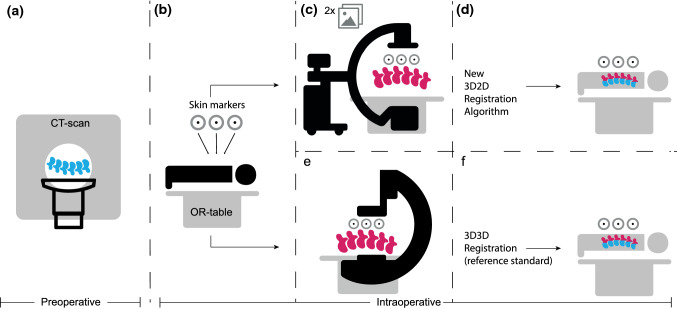
Schematic overview of how we could assess the Target Registration Error (TRE) between the two registration algorithms. **a** Baseline Computed Tomography (CT) scan of the subject in supine position. **b** The study subject is placed prone on the surgical table and the radiopaque adhesive skin markers are randomly placed on the back. **c** Two fluoroscopic images are acquired capturing the vertebrae and the marker model. **d** The 3D2D registration algorithm performs the registration offline. **e** Cone-beam CT scan is acquired capturing the same vertebrae and identical marker model. **f** The 3D3D registration algorithm (reference standard) performs the registration offline

The 3D2D registration algorithm’s accuracy was assessed by comparing it to a 3D3D registration based on a reference imaging device using cone-beam CT (CBCT) (Fig. 2). The CBCT was performed by a calibrated, motorized, ceiling-mounted, C-arm system installed in the surgical suite (Philips AlluraClarity FD20, Philips, Best, Netherlands).

The 3D3D registration was a rigid intensity-based registration that registered the 3D volume of each vertebra from the baseline CT scan to the position of the same vertebra as determined by the CBCT to correct for any local vertebral deformation regarding the 3D volume that had occurred between the baseline CT scan and the CBCT [12, 15]. After 3D3D registration and 3D2D registration, the 3D volumes of corresponding vertebrae were identical and because the marker model for both registrations was also identical, the registration accuracy of the 3D2D registration algorithm could be assessed (Fig. 2).

### Accuracy measurement

**Fig. 3:**
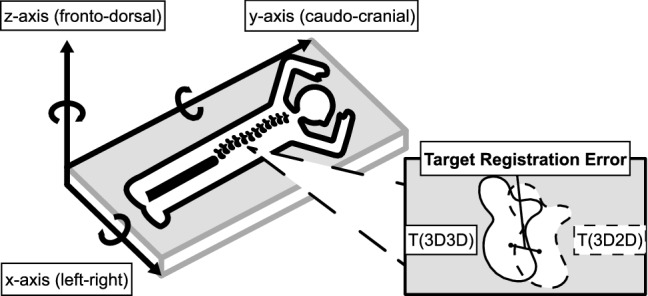
Schematic overview of the three-dimensional coordinate system that was used for calculating the Target Registration Error (TRE). The TRE was the three-dimensional distance in millimeters between the centers of corresponding vertebrae as registered by the two registration methods

The accuracy measure was the Target Registration Error (TRE) in millimeters. The TRE is a recommend measure to evaluate the accuracy of 2D to 3D registration [12] and is calculated by measuring the distance between similar points within corresponding vertebrae as registered by two registration methods in a 3D coordinate system [21]. The TRE was calculated between the centers of vertebral bodies (Fig. 3). The registration error was also visually inspected for every registration and was on sub-voxel level [12].

Additionally, although the TRE is a translational error measure, an indication of the rotational error was calculated. The rotational error was estimated by the TRE difference between two different points within one vertebra for which the TRE was separately calculated: if the 3D2D registration algorithm registered two different points in one vertebra with the same TRE (no TRE difference), it is unlikely that a rotational error occurred, but if the TRE differed between the two points, some rotational error had occurred. The location of the two points, the centers of the vertebral body and the left pedicle, were automatically determined in the model-based segmentation of the spine. We chose the centers of the vertebral body and the left pedicle because they are usually within the trajectory of a pedicle screw.

### Anatomical and radiologic parameters

Radiologic parameters included: the relative angulation between the fluoroscopic images, the detector-skin distance, the number of skin markers applied, and the captured level. Anatomical parameters included: the study subject and the anatomical region.

Fluoroscopic images were taken at different angles from the anterior–posterior (AP) position of the cadaveric specimen. The mobile C-arm always remained in neutral position toward the cranio-caudal direction (transversal plane). The mobile C-arm was manually rotated toward the latero-lateral direction (sagittal plane) to acquire fluoroscopic images in angles of − 45°, − 32°, − 30°, − 28°, − 20°, + 20°, + 28°, + 30°, + 32°, or + 45°. Pairs of fluoroscopic images with opposed angulations (e.g., − 20° and + 20°) were used for 3D2D registration resulting in relative rotation angle differences (RAD) of 40°, 56°, 60°, 64°, and 90° between the 2D images. The detector-skin distance was either 20 cm or 30 cm measured from AP position. The number of skin markers varied between seven to nine markers. Anatomical regions included three centered vertebrae in the thoracic (T4/T7/T10) and two in the lumbar spine (L1/L4). Each fluoroscopic image fully captured five vertebrae: a centered vertebra, and two adjacent vertebrae above and below. From the fluoroscopic images with L4 centered, only four vertebrae were registered because the algorithm did not register sacral vertebrae.

### Workflow of the experiment

All frozen cadaveric specimens underwent CT in a supine position. Before transfer to the operating room, each torso was thawed at room temperature for 72 h to allow for spine curvature changes and the shifting of vertebrae. In the operating room, the cadaveric specimens were placed in a prone position on the surgical table (representing a realistic surgical position). All subjects underwent identical test cases consisting of a combination of anatomical and radiologic parameters during which the 2D images were acquired.

**Fig. 4:**
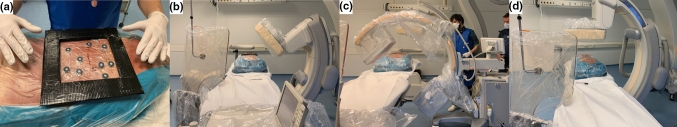
Workflow of a test case. **a** Study subject is placed prone on the surgical table and the adhesive skin markers are randomly placed within a frame of 15 × 15 cm. **b** A Cone-Beam Computed Tomography Scan (CBCT) is performed. **c** Fluoroscopic images are acquired. **d** Another CBCT is performed from the exact same position as the previous CBCT

At the beginning of each test case, the centered vertebra was identified through fluoroscopy. Then, skin markers were placed randomly on the back of each torso within a frame of 15 × 15 cm around the centered vertebra. First, a CBCT was performed with the fixed C-arm system. Subsequently, fluoroscopic images were obtained with the mobile C-arm from various angles depending on the test case. At last, an additional CBCT was performed from the exact same position as the previous. This cycle was repeated for every test case (Fig. 4).

The second CBCT was performed to confirm that neither the study subject nor the markers had moved between the first CBCT and fluoroscopic image acquisition to ensure that both the 3D2D and the 3D3D registration had registered vertebrae with an identical marker model. The two CBCTs were compared offline and only the first CBCT was used for 3D3D registration.

### Statistical analysis

The primary outcome was the TRE of which the median, interquartile range (IQR), outliers, and the maximum value were assessed.

Descriptive statistics were summarized for all parameters divided into groups. Bivariate analysis was performed with Spearman's rank correlation test for the continuous parameter RAD. Inter-group differences for the other parameters were assessed using the unpaired Wilcoxon rank-sum test for two groups and the Kruskal–Wallis test for more than two groups. Post-hoc analysis was conducted with a Bonferroni correction.

The parameters study subject, RAD, detector-skin distance, anatomical region, and captured level were included in a multiple regression analysis with backward elimination to determine parameters independently affecting the registration accuracy. Model assumptions were checked using histograms and quantile–quantile plots of residuals. The number of skin markers was not part of the multivariable analysis because this parameter was not assessed for every test case. Test cases with seven or nine markers were only assessed with a RAD of 60° and a detector-skin distance of 20 cm.

Additionally, an indication of the rotational error was calculated and the TRE was assessed for all three 3D axes to analyze the direction of the error in more depth [21].

*p *values of < 0.05 were used to denote statistical significance. TRE distributions were displayed with histograms and boxplots. Outliers were defined as the values above or below the upper or lower fences. The upper and lower fences represent values more and less than the 3rd and 1st quartiles, respectively, by 1.5 times the difference between the 3rd and 1st quartiles [22]. All statistical analyses were performed with R statistical software (R, version 4.0.3).

## Results

**Fig. 5:**
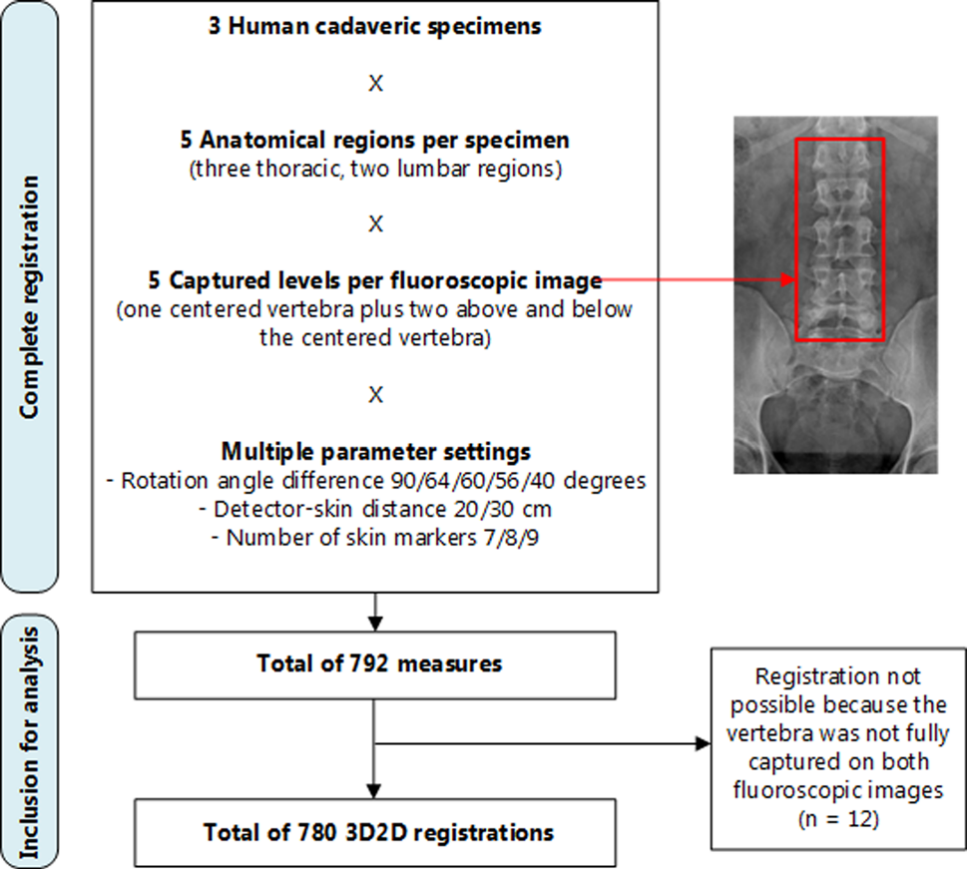
Flowchart of 3D2D registrations

In total, the 3D2D registration algorithm successfully registered 780 vertebrae. Twelve vertebrae (two thoracic and ten lumbar) could not be registered as they were not fully captured on both fluoroscopic images. The unsuccessful registrations could not be corrected because the 3D2D registration was performed offline at a different moment (Fig. 5).

**Fig. 6:**
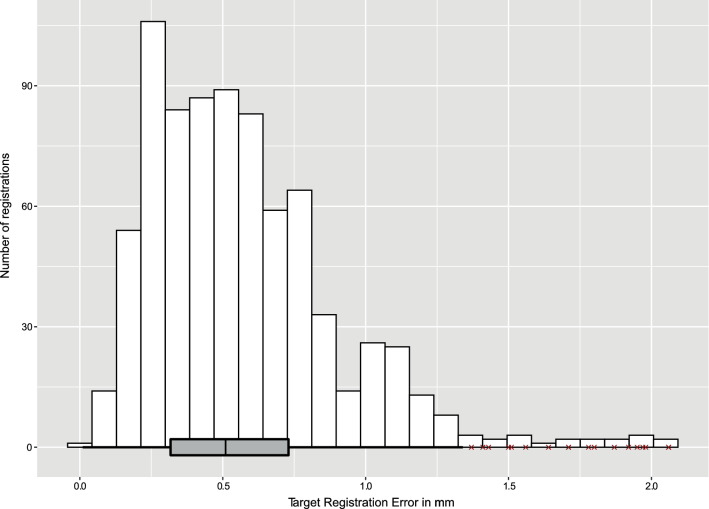
Distribution of the TRE in millimeter (*n* = 780). The median TRE was 0.51 mm [IQR 0.32–0.73 mm]. All 18 outliers were performed with a rotation angle difference of 40°, 12 were in thoracic vertebrae, 11 in subject B and 7 in subject A

The algorithm had a median TRE of 0.51 mm [IQR 0.32–0.73 mm], and a maximum TRE of 2.06 mm. The algorithm had eighteen outliers, which all occurred in registrations performed with a RAD of 40° and in the two subjects with a larger waist circumference: subject A had a circumference of 121 cm and subject B had a circumference of 96 cm (Fig. 6) (Table 1 shows the waist circumference of the subjects).

In bivariate analysis, no inter-group difference was observed regarding the TRE for the parameter captured level (*p* = 0.473). The parameters study subject (*p* < 0.001), anatomical region (*p* = 0.003), detector-skin distance (*p* = 0.007) and number of markers (*p* = 0.021) all showed statistically significant inter-group differences (Table 2). Post-hoc analysis revealed that the median TRE of subject C was lower than that of subjects A and B. The registrations with eight skin markers had a higher median TRE than the registrations with seven and nine markers.

**Table 2:** Descriptive statistics of all parameters on TRE

	Number of registrations	Median TRE in mm (± IQR)	Statistical test	*p* value
*Study subject*				
Subject A	259	0.56 [0.33–0.75]	Kruskal–Wallis	< 0.001
Subject B	259	0.53 [0.38–0.81]		
Subject C	262	0.45 [0.27–0.64]		
*Anatomical region*				
Thoracic	493	0.50 [0.29–0.71]	Mann–Whitney U	0.003
Lumbar	287	0.53 [0.38–0.80]		
Rotation angle difference*				
40°	143	0.79 [0.52–1.12]	Spearman’s rank correlation	< 0.001 (*ρ* = − 0.34)
56°	141	0.51 [ 0.38–0.74]		
60°	283	0.47 [0.32–0.69]		
64°	141	0.49 [0.27–0.64]		
90°	72	0.33 [0.22–0.45]		
*Detector-skin distance from AP position*				
20 cm	499	0.49 [0.31–0.69]	Mann–Whitney U	0.007
30 cm	281	0.56 [0.33–0.79]		
*Captured level*				
Centered vertebra	165	0.48 [0.28–0.75]	Mann–Whitney U	0.473
One level from centered vertebra	328	0.49 [0.33–0.71]		
Two levels from centered vertebra	287	0.53 [0.32–0.77]		
Number of skin markers**				
7 markers	70	0.48 [0.35–0.63]	Kruskal–Wallis	0.021
8 markers	72	0.6 [0.32–0.92]		
9 markers	71	0.42 [0.29–0.57]		

*To calculate the median and IQR, rotation angle differences were divided into groups according to the amount of degrees; however, to assess the correlation with the TRE, the rotation angle difference was considered a continuous variable

**For a direct comparison, only the registrations (*n* = 213) with a rotation angle difference of 60° and a detector distance-skin of 20 cm were included for this analysis. All other registrations (*n* = 639) were performed with 8 markers and were performed with a different relative angulation and/or detector-skin distance

Underlined *p*-values indicate a stastically significant difference (*p* < 0.05)

The TRE was best predicted by the multiple regression model with RAD, anatomical region, and study subject as predictors (F-statistic (4, 491) = 16.22, *p* < 0.001, adj *R*^2^ = 0.11). The parameter RAD was the most important predictive factor with a standardized coefficient *β* of − 0.41 (*p* < 0.001), i.e., the larger the RAD (with a current maximum of 90°), the lower the TRE. The 3D2D registration algorithm was more accurate in thoracic vertebrae (*p* = 0.004) and subject C (*p* = 0.003). The standardized coefficients *β* were small for both thoracic vertebrae (− 0.10), and subject C (− 0.12) (Table 3).

**Table 3:** Multiple regression analysis of parameters influencing the accuracy of 3D2D registration after backward selection

Independent variable	Unstandardized coefficients		Standardized coefficients *β*	*p *value
	B	Standard error		
Intercept	1.24	0.06		
Rotation angle difference	− 0.01	0.00	− 0.41	< 0.001
*Anatomical region*				
Thoracic	− 0.07	0.03	− 0.10	0.004
Lumbar	Reference value	Reference value	Reference value	Reference value
*Study subject*				
Subject A	Reference value	Reference value	Reference value	Reference value
Subject B	0.06	0.3	0.08	0.06
Subject C	− 0.09	0.03	− 0.12	0.003

Adjusted *R*^2^ = 0.11, F-statistic (4, 491) = 16.22, *p *value of the model ≤ 0.001

Because the registration accuracy of the algorithm increased (thus, a lower TRE) with the increase in the RAD (larger angulations), the registration accuracy was explored by cumulatively excluding registrations with a RAD from small to large. Excluding registrations performed with a RAD of 40° (*n* = 143) resulted in a median TRE of 0.47 mm [IQR 0.30–0.67 mm] for the remaining 637 registrations. Additionally excluding registrations performed with a RAD of 56° (*n* = 141) resulted in a median TRE of 0.45 mm [IQR 0.28–0.63 mm] for 496 registrations, and subsequently excluding registrations performed with a RAD of 60° (*n* = 265) resulted in a median TRE of 0.42 mm [IQR 0.25–0.59 mm] for 213 registrations. The median TRE was 0.33 mm [IQR 0.22–0.45 mm] for the 72 registrations performed with a RAD of 90°. The registrations in thoracic vertebrae (*n* = 493) had a median TRE of 0.50 mm [IQR 0.29–0.71 mm] and the registrations in lumbar vertebrae (*n* = 287) a median of 0.53 mm [IQR 0.38–0.80 mm].

**Fig. 7:**
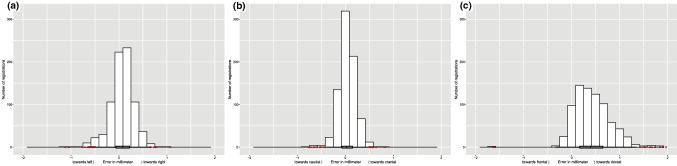
Distributions of the TRE in millimeter for all three dimensions (*n* = 780). The largest TREs were in the *z*-axis and were skewed toward the dorsal side of the patient. **a** The TRE in millimeter regarding the *x*-axis. The median TRE was 0.08 mm [IQR − 0.07 to 0.22 mm]. In total, 31 outliers occurred, which were in the registrations performed with rotation angle differences of 40° (*n* = 11), 56° (*n* = 2), 60° (*n* = 7), 64° (*n* = 8), and 90° (*n* = 3), thoracic vertebrae (*n* = 11), subject A (*n* = 14) and subject B (*n* = 17). **b** The TRE in millimeter regarding the *y*-axis. The median TRE was 0.03 mm [IQR − 0.07 to 0.15 mm]. In total, 19 outliers occurred, which were in the registrations performed with rotation angle differences of 40° (*n* = 9), 56° (*n* = 4), 60° (*n* = 2), 64° (*n* = 2), and 90° (*n* = 2), thoracic vertebrae (*n* = 1), subject A (*n* = 15), subject B (*n* = 2) and subject C (*n* = 2). **c** The TRE in millimeter regarding the *z*-axis. The median TRE was 0.39 mm [IQR 0.17–0.65 mm]. In total, 14 outliers occurred, which were in the registrations performed with a rotation angle difference of 40° (*n* = 14), thoracic vertebrae (*n* = 12), subject A (*n* = 7) and subject B (*n* = 7)

**Fig. 8:**
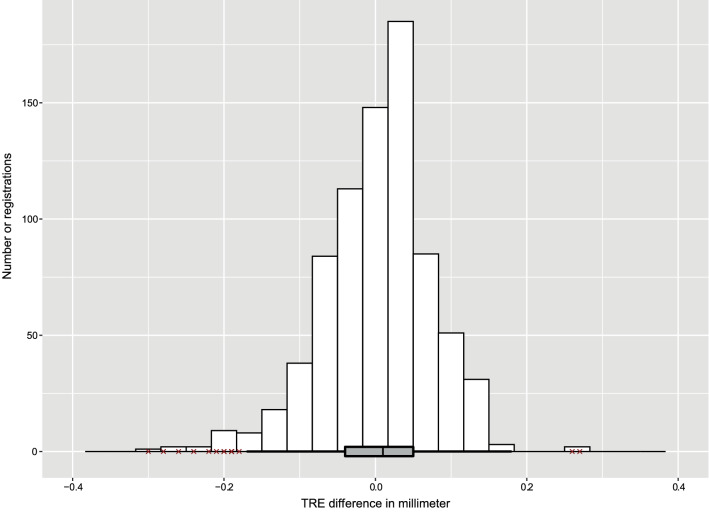
The TRE difference between two points (left pedicle and center of vertebral body) within one vertebra (*n* = 780). The median TRE difference was 0.01 mm [IQR − 0.04 to 0.05 mm]. In total, 17 outliers occurred, which were in the registrations performed with rotation angle differences of 40° (*n* = 3), 56° (*n* = 3), 60° (*n* = 8) and 64° (*n* = 3), thoracic vertebrae (*n* = 12), subject A (*n* = 6), subject B (*n* = 3) and subject C (*n* = 8)

Translational errors for the *x*-axis (left–right direction) and the *y*-axis (caudo-cranial direction) were small, and normally distributed. The algorithm was the least accurate on the *z*-axis (fronto-dorsal direction): the TRE was skewed toward the dorsal side of the study subjects with a median of 0.39 mm [IQR 0.17–0.65 mm] (Fig. 7). The TRE difference between two points within one vertebra (rotational error) was normally distributed with a median of 0.01 mm [− 0.04 to 0.05 mm] (Fig. 8).

## Discussion

### Key results

We assessed the accuracy of a new 3D2D registration algorithm based on a non-invasive skin marker model to be used for navigated spine surgery. The algorithm registered five adjacent vertebrae in the thoracic and lumbar spine from three human cadaveric specimens. When all 780 registrations were included, the algorithm had a median TRE of 0.51 mm [IQR 0.32–0.73 mm] and a maximum TRE of 2.06 mm. The algorithm registered all five adjacent vertebrae with similar accuracy.

### Interpretation

Few navigation systems for spine surgery using an integrated 3D2D registration algorithm are commercially available, for example, the ExcelsiusGPS by GlobusMedical and the Mazor X Stealth Edition by Medtronic [13]. The registration setup of the algorithm under study may be more simple and intuitive than the existing algorithms because no bone-mounted or table-mounted instruments are needed, nor is any equipment required for calibrating and tracking the C-arm. The non-invasive skin markers do not require surgeons to make additional incisions for registration instruments and, because the C-arm does not have to be tracked or calibrated, allow for quick registration and re-registration with minimum disruption of the routine surgical workflow. In addition, surgeons can easily replace the markers to register other vertebrae and expand the vertebral levels they can treat during one procedure.

Other non-invasive 3D2D registration algorithms for spine surgery serve a different intended clinical application. For instance, some algorithms aim to automatically label vertebrae at the start of surgery to prevent wrong-level interventions [23, 24], while other algorithms aim to intraoperatively verify the 3D position of pedicle screws [25–27]. Our non-invasive 3D2D registration algorithm aims to provide surgeons navigation for interventions such as pedicle screw insertion. However, in future, applications from existing algorithms may be added to the algorithm under study, such as automatically labeling vertebrae to prevent wrong-level surgery and 3D2D registration for intraoperatively verifying screw positions.

When the 3D2D registration algorithm is integrated into a complete navigation system, the system will contain new elements such as a camera unit and specific surgical tools that could potentially affect the overall precision. Based on the accuracy of available navigation systems, 3D2D navigation may be considered feasible for clinical practice if no major outliers occur and, if pedicle screws breach the pedicle wall, the breach is less than 2 mm [7, 28, 29]. When including all registrations, 95% of the TREs were below 1.16 mm but outliers existed up to 2.06 mm. Registrations with a RAD of 40° were the least accurate and responsible for all outliers. When excluding registrations with a RAD of 40° (thus keeping RADs between 56° and 90°), 95% of the TREs were below 1.02 mm and the maximum TRE was 1.28 mm. Although future studies should explore the exact boundaries of the algorithm, a RAD of 40° may be too small for safe application of 3D2D navigation. Commercially available systems often require a specific RAD of 90° for 3D2D registration [8, 13]. The practical impact may be small, but if the current algorithm remains accurate with RADs between 56° and 90°, it may provide a more flexible and safer future workflow. Registration does not require one specific angulation between the 2D images and less movement of the C-arm reduces the chance of breaking the sterile field.

The algorithm performed best in subject C and in thoracic vertebrae. Subject C had less attenuating tissue because of a smaller waist circumference and the thoracic vertebrae had less attenuating tissue than the lumbar vertebrae, which improved the quality of the fluoroscopic images [30, 31]. Also, the CT scans of the subjects with a larger waist circumference had a relatively larger voxel size. Because each CT scan had a field of view that contained the whole study subject and was not limited to the vertebral column, subject A had almost twice the voxel size of subject C. However, during pre-experimental test runs using a phantom, changing the field of view and, therefore, the relative voxel size did not alter the algorithm's accuracy. Still, if obesity becomes extreme and bone density also decreases severely, the algorithm’s accuracy might compromise patient safety [8].

Bivariate analysis revealed that the algorithm was less accurate with eight skin markers than seven or nine markers, but a logical explanation is lacking. The algorithm needs a minimum of five skin markers for registration, and during the experiment, all markers were randomly applied within the same frame of 15 × 15 cm. If registration accuracy depends on the number of markers used, one would expect the registration accuracy to also decrease for nine markers indicating that there is a maximum number of markers for accurate registration. Future experiments should include different numbers of markers in multivariable analysis to assess if registration accuracy directly depends on the number of markers used.

In-depth analysis of translational errors showed that most errors occurred in the *z*-axis (toward the dorsal side of the patient). The algorithm had the lowest capture range over this axis because the study subjects were prone on the surgical table and the maximum angulation of the mobile C-arm was 45° from AP toward the lateral side of the study subject. For example, all outliers were registrations with the lowest fluoroscopic capture range (angulation of 40°). One could argue that an error in this direction is less crucial for pedicle screw placement as critical structures like the spinal cord are located medially (*x*-axis). However, a high registration accuracy in all axes becomes necessary if 3D2D navigation is applied for different purposes, such as navigated vertebral biopsy. Future studies may expand the latero-lateral angulations and experiment with angulation in other directions, such as cranio-caudal angulations, to optimize the registration accuracy [32].

In the current study, we only evaluated CT scans with parallel slices of 0.67 mm. The algorithm was developed to work with any CT scan vendor, so it can easily integrate in clinical practice. The algorithm would integrate even more easily if it can also cope with various scan protocols. The current algorithm interpolates the unknown values of pixels lying between slices with a known value to generate a complete 3D volume, but the present study did not assess at what slice thickness it becomes inaccurate. In the supplementary data, we explored the algorithm’s accuracy using post-processed baseline CT scans with a slice thickness up to 5.0 mm. The supplementary figures indicate that the algorithm remains similarly accurate with slice thicknesses up to 2.0 mm but the exact limits should be explored using a higher number of scans (Supplement 1).

## Limitations

A drawback of 3D2D registration, in general, might be the assumption that, at the time of intraoperative registration, the 3D volume of vertebrae has not altered since the baseline CT scan. Although the algorithm registered each vertebra separately and accounted for positional changes of individual vertebrae up to 5° rotation around the *x*-axis and translations up to 2 mm over the *z*-axis, it may be necessary to maintain a short interval between the baseline CT and operative treatment regarding, for instance, the 3D volume of collapsed vertebrae may change.

Another limitation was that the rotational error was not exactly calculated but estimated by the TRE difference between two points in a single vertebra representing the trajectory of a pedicle screw. The maximum difference was 0.30 mm, and apart from 17 outliers, all values were smaller than 0.19 mm, suggesting that the rotational error was small. Still, future studies need to assess the exact rotational error, for example, by evaluating the angle deviation from the planned path of a navigationally inserted pedicle screw.

Furthermore, the circumstances of the current experiments differed from normal circumstances because the subjects were frozen at the time of baseline CT and the 2D images were acquired in a thawed state. Also, the 3D2D registration was performed offline on a different moment than the 2D image acquisition. As a result, the unsuccessful registration of twelve vertebrae could not be restored but it was unlikely that these would have changed the study results. Test cases using two different 2D images to register the same vertebrae were accurate. Eventually, the navigation system will not be limited by unsuccessful registrations because the registration is executed directly in the operating room, obliging the surgeon to re-register vertebrae when initial registration fails.

Integrating the algorithm into a navigation system also generates new challenges, such as intraoperative 2D image acquisition and registration time. For example, during surgery, between the acquisitions of the two 2D images, the algorithm will require a patient's breath-hold (under anesthesia) so that the marker model's relative position to the vertebrae remains the same. A breath-hold under anesthesia urges the surgical team to acquire the 2D images in a short time and may be considered a complicated intervention. Another challenge for clinical implementation is keeping registration time as short as possible, preferably under a few minutes. In the current study, we did not calculate the exact registration time but the algorithm performed all registrations on a regular computer in up to a few minutes. No optimizations for limiting registration time have been implemented yet.

### Intended workflow of 3D2D navigation

**Fig. 9:**
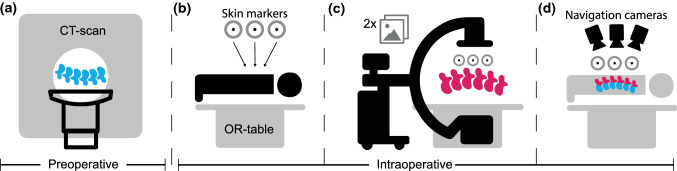
Intended workflow of 3D2D navigation. **a** A preoperative CT scan is acquired (in supine position). **b** Intraoperatively, the radiopaque adhesive skin marker are applied on the back of the patient. **c** Intraoperatively, 3D2D registration is performed with the preoperative CT data, and two fluoroscopic images capturing the marker model and vertebrae. **d** After 3D2D registration, the marker model is registered to optical cameras track the marker model, thus indirectly tracking the vertebrae, to allow for intraoperative navigation

Spinal navigation tracks the patient's bony anatomy (such as vertebrae) and compatible surgical tools in 3D during surgery. Figure 9 shows our concept of 3D2D navigation: optical cameras indirectly track the patient's vertebrae by tracking a marker model applied to the back of the patient. The marker model consists of a pattern of hybrid adhesive skin markers (radiopaque and optical).

First, the 3D volume of vertebrae is obtained before the surgical procedure using data from a previous CT scan (without markers). Then, in the operating room, after the incision, the adhesive skin markers are applied when the patient is prepared to undergo pedicle screw insertion. The marker model is the reference to relate 3D2D registration (fluoroscopic space) to optical navigation (navigated space). The 3D2D registration algorithm registers the reconstructed 3D volume of vertebrae from the pre-existing CT scan to their intraoperative position in two fluoroscopic 2D images. The two fluoroscopic images must both fully capture the marker model and the vertebrae that must be registered so that the navigation system can relate fluoroscopic space to the navigated space. Using optical cameras, the navigation system indirectly tracks the position of the registered vertebrae based on the marker model (Fig. 9)*.*

## Conclusion

We studied the accuracy of a new 3D2D registration algorithm based on a non-invasive skin marker model. The algorithm registered five adjacent vertebrae in the thoracic and lumbar spine, and showed a maximum target registration error of approximately 2 mm. All five adjacent vertebrae were registered with similar accuracy. To further evaluate its potential for navigated spine surgery, the algorithm may now be integrated into a complete navigation system.

## Supplementary Information

Below is the link to the electronic supplementary material.

Supplementary file1 (DOCX 287 kb)

## Data Availability

Not available.

## Code availability

R-code available upon request with the corresponding author.
