# Supplementary material for: Assessing the accuracy of a new 3D2D registration algorithm based on a non-invasive skin marker model for navigated spine surgery
Source: Int J Comput Assist Radiol Surg. 2022 Aug 20;17(10):1933–45. doi: 10.1007/s11548-022-02733-w (PMC9468112; doi:10.1007/s11548-022-02733-w)
Supplement: Supplementary file 1 — Supplementary file1 (DOCX 287 kb) [file 11548_2022_2733_MOESM1_ESM.docx]

**Supplement 1**

Figures of the TRE in mm for multiple slice thicknesses of the same baseline CT scan. The same test case for each study subject was used consisting of a rotation angle difference of 30 degrees, detector-skin distance of 20cm and the application of eight adhesive skin markers. The centered vertebra was L1. Slice thicknesses were chosen according to various scan protocols of the hospital including: parallel slices of 0.67mm, 1.0mm, 1.5mm and 2.0mm, a slice thickness of 0.90mm with an increment of 0.70mm, and a slice thickness of 5.0mm with an increment of 4.0mm. The TRE of the latter is not displayed in the figures due to the high values.

**Supplemental Figure 1**

1. The TRE for the vertebra two levels above L1 (T11). The TRE using a slice thickness of 5.0mm was 0.97mm for subject A, 1.12mm for subject B, 93.73mm for subject C.


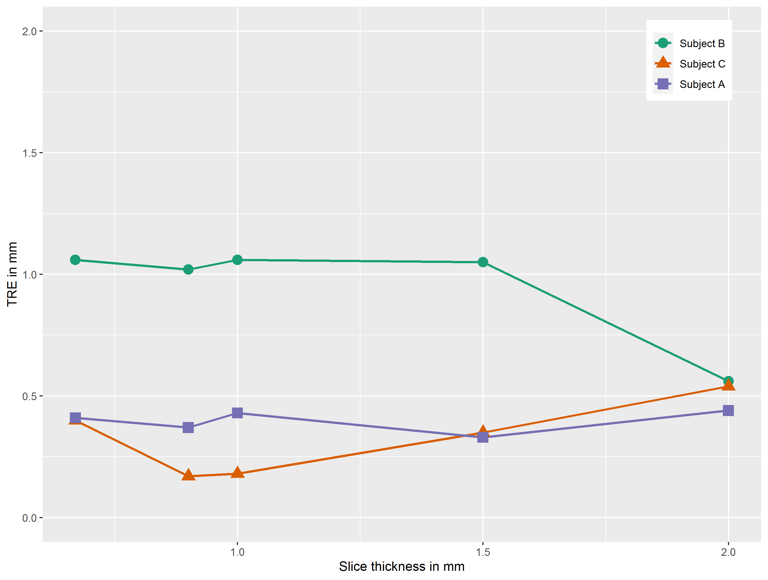


1.
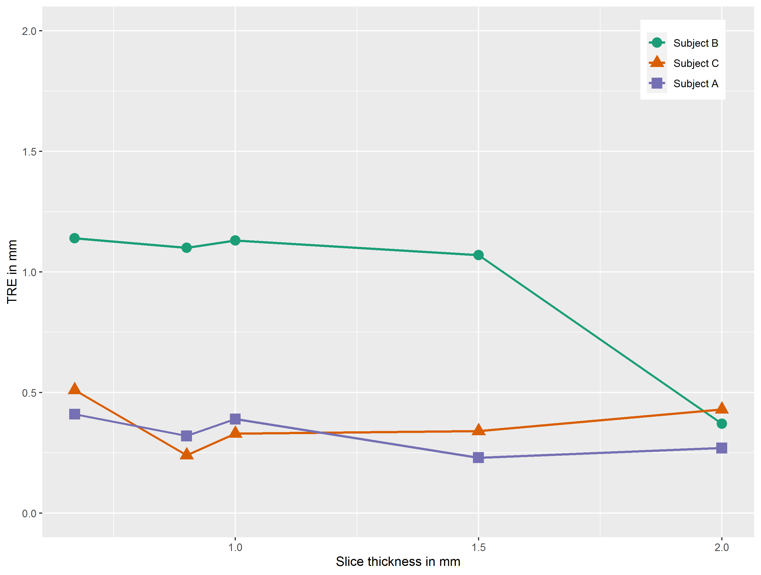
The TRE for the vertebra one level above L1 (T12). The TRE using a slice thickness of 5.0mm was 0.49mm for subject A, 1.27mm for subject B, 0.96mm for subject C
2.
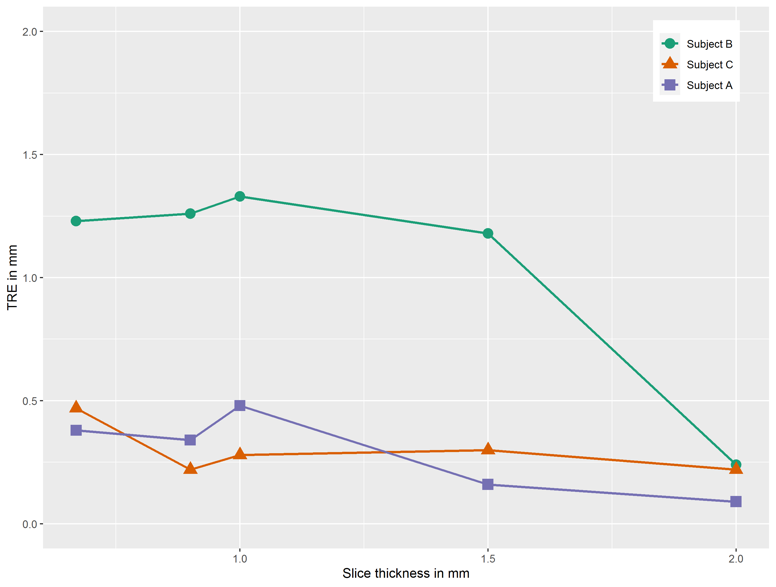
The TRE for the centered vertebra (L1). The TRE using a slice thickness of 5.0mm was 0.26mm for subject A, 1.27mm for subject B, 68.92mm for subject C
3.
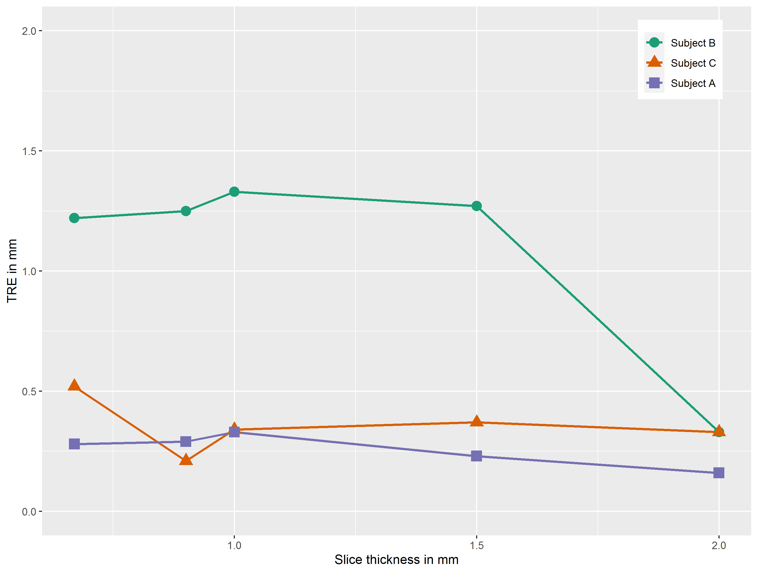
The TRE for the vertebra one level below L1 (L2). The TRE using a slice thickness of 5.0mm was 0.76mm for subject A, 1.76mm for subject B, 0.73mm for subject C
4. The TRE for the vertebra two levels below L1 (L3). The TRE using a slice thickness of 5.0mm
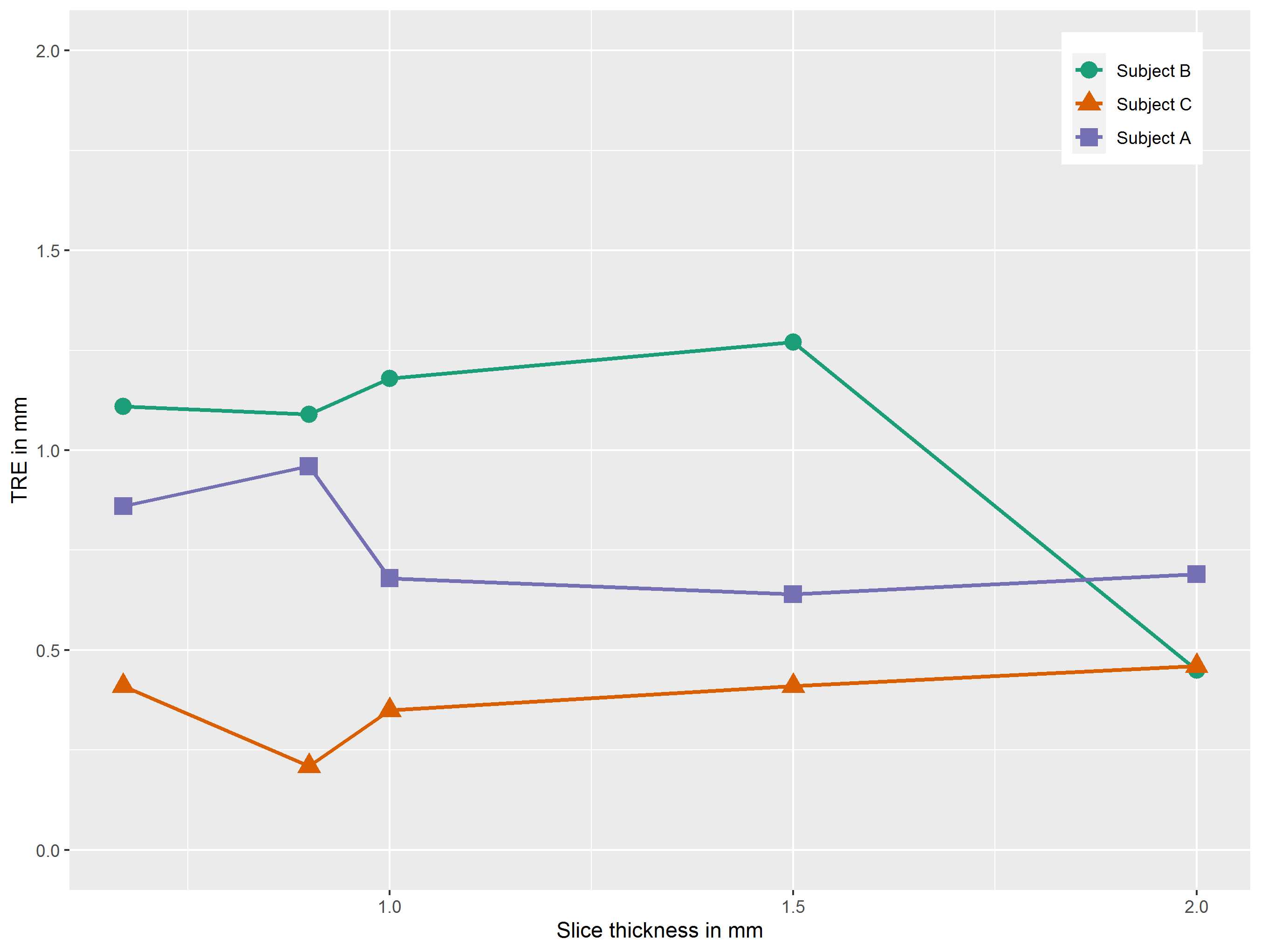
was 1.14mm for subject A, 52.53mm for subject B, 1.81mm for subject C
